# Beyond nutritional immunity: immune-stressing challenges basic paradigms of immunometabolism and immunology

**DOI:** 10.3389/fnut.2025.1508767

**Published:** 2025-02-12

**Authors:** Edmund K. LeGrand

**Affiliations:** Biomedical and Diagnostic Sciences, College of Veterinary Medicine, University of Tennessee, Knoxville, TN, United States

**Keywords:** immunometabolism, nutritional immunity, host-pathogen interactions, glycolysis, glucose, lactic acid, heat, oxidative burst

## Abstract

Pathogens have the well-known advantage of rapid evolution due to short generation times and large populations. However, pathogens have the rarely noted disadvantage of the vulnerability to stress involved in proliferation as well as being localized. Presented here are numerous new paradigms in immunology, and especially immunometabolism, which are derived from examining how hosts capitalize on pathogen vulnerabilities to stress. Universally, proliferation requires both resources and synthesis, which are vulnerable to resource-limiting stress and damaging/noxious stress, respectively. Pathogens are particularly vulnerable to stress at the time when they are most threatening—when they are proliferating. Since immune cells actively controlling pathogens (effector cells) typically do not proliferate at infected sites, there is a “stress vulnerability gap” wherein proliferating pathogens are more vulnerable to any type of stress than are the attacking effector cells. Hosts actively stress vulnerable proliferating pathogens by restricting resources (resource-limiting stress) and generating noxious waste products (damaging/disruptive stress) in a fundamental defense here-in termed “immune-stressing.” While nutritional immunity emphasizes denying pathogens micronutrients, immune-stressing extends the concept to restricting all resources, especially glucose and oxygen, coupled with the generation of noxious metabolic products such as lactic acid, reactive oxygen species (ROS), and heat to further harm or stress the pathogens. At present much of the field of immunometabolism centers on how nutrition and metabolism regulate immune function, a central feature being the inefficient use of glucose via aerobic glycolysis (with much lactate/lactic acid production) by effector immune cells. In contrast, immune-stressing emphasizes how the immune system uses nutrition and metabolism to control infections. Immune-stressing addresses effector cell glycolysis *at the infected site* by noting that the high uptake of glucose linked with high output of lactic acid is an ideal double-pronged stressor targeting proliferating pathogens. Once the basic vulnerability of pathogen proliferation is recognized, numerous other paradigms of immunometabolism, and immunology as a whole, are challenged.

## 1 Introduction

This conceptual analysis explores the logical consequences of a fundamental principle, that the process of proliferation, requiring resources and synthesis, is vulnerable to stress from limitation of resources (nutrients, including oxygen) and from disruptive or damaging stress caused by noxious agents. In host-pathogen conflicts there are well-known advantages that pathogens have because of rapid proliferation and large population size permitting rapid evolution, most notably in antibiotic resistance, even within an individual host. However, only rarely noted are the pathogen disadvantages of generally being localized, hence safely permitting application of intense stress by the host, and of having to proliferate (1, 2). This neglect is puzzling since the logical consequences explored here provide crucial insights that challenge or provide numerous new paradigms of immunometabolism and even immunology. Table 1 lists 12 such novel concepts. Most of the analysis will address immunometabolism and its foundations as they relate to the advantages of effector cells *in direct conflict with pathogens,* which are vulnerable due to their localization and proliferation.

**Table 1:** Paradigms derived from immune-stressing (i.e., stressing vulnerable localized proliferating pathogens by restricting resources and generating noxious waste products).

Effector immune cells actively create much of the stressful conditions at infected sites. Since this preferentially harms the proliferating pathogens, creating this non-specific stress is one of the key functions of effector cells (2).
Effector cells typically do not proliferate at infected sites because it is too stressful. Were they to proliferate there, they would lose their advantage of using stress to preferentially harm the pathogens (2).
Nutritional immunity, the restriction of micronutrients to pathogens, is overshadowed by “extended” nutritional immunity, the restriction of all nutrients, including oxygen, to the pathogens (2).
The restricted nutrients can be converted to noxious products, acting as damaging stressors to harm the more vulnerable pathogens (2).
High uptake of glucose and glutamine by effector cells *at infected sites* does not reflect the functional needs of the cells, instead functioning to deprive pathogens of nutrients (2).
The metabolic needs of effector cells *when confronting pathogens* cannot be determined by uptake of resources/nutrients or output of metabolic products (any more than the metabolic needs of adipose cells can be determined by measuring lipid uptake).
The enhancement of glycolysis of effector cells not only depletes glucose, but also generates noxious acidity from lactic acid (2).
Acetic acid, found in very high concentrations at infected sites (55), is likely another host-generated noxious stressor.
Oxygen is actively depleted from infected sites and is converted to ROS as neutrophils move toward inflamed sites (56). (Oxygen depletion impairs pathogens’ ability to oxidize lipids, amino acids, lactate, and acetate for fuel).
Localized heat generated from the oxidative burst is likely to be the main source of heat stress applied to pathogens, with the systemic heat of fever a lesser contributor (2, 29).
The oxidative burst harms pathogens not only by ROS (a noxious product), but also by oxygen depletion (a resource) and local heat generation (a noxious product).
Immunometabolism should emphasize how the immune system uses metabolism to control pathogens, in addition to emphasizing how metabolism controls the immune system.

Although there has long been interest in the broad intersection of nutrition and metabolism with immune responses, the term “immunometabolism” began being used only in the past decade (3, 4). Immunometabolism has come to emphasize that metabolic processes and the nutritional microenvironment regulate immune cells, especially effector cells whose function is to control pathogens (5–11). Pathogens can be either extracellular (including tumor cells as endogenous pathogens) or intracellular. Infected host cells manipulated by their intracellular pathogens are themselves pathogens and are treated as such by the immune system. The pathogens considered in this conceptual analysis are those that proliferate at the infected (or tumor) site, notably bacteria, protozoa, fungi, viruses, and tumor cells. Specifically excluded are metazoan parasites not proliferating in the tissues where they may occur. Although the function of the immune system is to control pathogens, the field of immunometabolism generally fails to consider pathogens except as competitors of immune cells for nutrients. Nutrient-hungry pathogens are considered a problem since very low levels of resources or very high levels of metabolic wastes impair effector cells’ functionality. In other words, in the standard view the effector cells and pathogens are nearly equally matched in terms of resource depletion. However, here it is argued that overlooking the vulnerability of proliferation to stress by localized pathogens (2) has led the field of immunometabolism astray by focusing on metabolic control of immune responses rather than on the immune system’s core function of controlling pathogens.

A central theme of immunometabolism is effector cells’ enhanced use of glucose for ATP production through aerobic glycolysis (with high lactate production) rather than relying predominantly on mitochondrial OXPHOS. While long recognized for neutrophils (12, 13) and monocyte-macrophages (M1 macrophages) (14), this glycolytic preference and high nutrient uptake, particularly of glucose and glutamine (15), has been found to extend to essentially all immune cells that are involved in directly controlling pathogens. Effector cells’ relative preference for glycolysis extends beyond neutrophils and M1 macrophages to include dendritic cells, effector T cells (cytotoxic and helper), natural killer cells, B lymphocytes (3), and even to platelets activated for clotting (16), which helps control pathogens (17, 18). This glycolytic preference, even in the presence of oxygen, occurs not only during cell proliferation and synthesis at distant low-stress sites, but also during activation for migrating toward and confronting the pathogens at infected sites. This confrontation with the pathogens is the phase of effector cell life history focused on in this conceptual analysis. The preference for glycolysis *at infected sites* is not intuitive since aerobic glycolysis generates only 2 ATPs per glucose molecule, while exporting most of the energy value of glucose as lactate/lactic acid, rather than generating the additional 36 ATPs theoretically possible from OXPHOS. Besides this apparent wastefulness of glucose, the large nutrient uptake by activated effector cells is often interpreted as indicating high nutrient needs for the apparently high costs of fighting pathogens (19, 20).

The interpretation of these and other experimental findings has become paradigmatic in immunometabolism. In contrast, this paper describes the host defense strategy of “immune-stressing”—actively making the infected site stressful to preferentially harm the more vulnerable proliferating pathogens. This strategy provides a simple and logical evolutionary-based alternative interpretation of many experimental findings, thereby challenging many of the standard principles of immunometabolism. Most notable are the need to consider the pathogen vulnerabilities to metabolic stress, to reconsider the function of aerobic glycolysis for effector cells confronting pathogens, and to recognize that the oxidative burst at the effector cell surface serves to harm pathogens by not only generating noxious reactive oxygen species (ROS), but also by depleting oxygen and by generating noxious localized heat, a long-ignored metabolic product.

## 2 Fundamentals of immune-stressing

Immune-stressing is an innate host defense strategy of applying stress (harm or possible harm), which preferentially affects proliferating pathogens more than the non-proliferating host cells (2). It is a fundamental principle relating to stress of any kind, and it has relevance beyond host-pathogen interactions to include cancer therapy, military strategy, international relations, finance, and more.

### 2.1 Universally, proliferation is especially vulnerable to stress

Proliferation requires resources, both for cellular components/materials and for fuel/energy. Resource-limiting stress is typically of slow onset. Proliferation also requires synthesis, which entails the precise manipulation of materials, usually to make more complex structures. Synthesis is vulnerable to damage or disruption from noxious stressors, often of rapid onset. That proliferation requires resources is a simple arithmetic principle. That synthesis is especially vulnerable can be viewed as a variant of the second law of thermodynamics, that systems tend toward disorder. Consequently, it takes effort to create ordered structures and even more effort in the face of disruptive or damaging conditions.

The universality of the principle is exemplified in the building of a house (2). It requires a complete set of materials along with labor (energy), thus being subject to resource-limiting stress. The process of handling and assembling the building materials (synthesis) requires low stress (hence predictable) conditions. However, the wind stress of a hurricane makes it very difficult to handle the materials and to assemble them properly. Additionally, an unfinished house is especially prone to damaging wind gusts and water damage unless extra effort is taken to secure this intermediate structure. This fundamental vulnerability of proliferation to stress applies to cellular processes in both hosts and pathogens.

In terms applicable to infections, the proliferation of an infective inoculum of a thousand bacteria to become a million requires at least a 1,000-fold increase in resources. The 1,000-fold population increase also requires essentially this much proliferation over about 10 generations (10^3^ ≈ 2^10^), each replication involving the delicate synthesis of precisely constructed molecules of nucleic acids and innumerable proteins and their highly choreographed interactions.

### 2.2 Infections start out localized

The localization of pathogens allows the host to apply much more intense stress than would be safe to apply diffusely. Extreme localization within phagolysosomes allows for especially intense application of stressors such as ROS, severe pH changes, very low or high metal ion concentrations (21), and presumably heat (22, 23), each of which would be lethal to the host if applied systemically. Essentially the same stressors of less intensity occur extracellularly at the infected sites. Systemically, similar stressors of much lower intensity occur as part of the acute phase response (24), permitting distant host cells to participate in pathogen control by supporting the stress gradient as well as mildly increasing the stress levels for pathogens that escape localization (2).

### 2.3 Pathogens typically must proliferate to be pathogenic

In contrast to most pathogens (including tumor cells and infected cells producing pathogens), most host tissues have limited immediate needs for proliferation. Although effector cells are essential at infected sites, they proliferate at distant low-stress locations, notably bone marrow and lymphoid tissues. It is notable that M1 (monocyte-derived) macrophages typically do not proliferate locally at infected sites, while M2 (tissue resident) macrophages involved in controlling metazoan parasites and promoting tissue repair do proliferate locally (25). It is argued that it is no coincidence that effector cells typically do not proliferate at infected sites because it is too stressful there, and that infected sites are stressful in part because (as proposed by immune-stressing) a function of effector cells is to actively create stress there (2).

### 2.4 The “stress vulnerability gap”

The “stress vulnerability gap” is the difference in relative vulnerability between local proliferating pathogens and the local host cells, particularly the effector cells. The host can capitalize on the stress vulnerability gap by increasing the localized stress, which can involve resource-limiting stress and/or damaging/noxious stress. Since much of the stress is actively host-induced as an immune function (2), it is not surprising that effector cells evolved to function best in somewhat stressful conditions, as noted for decreased oxygen (26), increased acidity (27, 28), and heat (29). Most cancer therapy is based on the increased vulnerability of proliferation to stress, while also taking advantage of localization of the tumor to permit applying more intense stress where feasible (with excision being the epitome of intense therapeutic stress).

As noted, metazoan parasites in tissues typically do not proliferate locally and thus are not subject to this stress vulnerability gap. Metazoan parasites are typically controlled with Type 2 immune responses, with eosinophils as specialized effector cells and with fibroblast proliferation and collagen synthesis. Especially interesting are the exceptions to the generalizations that (a) infections tend to start out localized, (b) that pathogens need to proliferate to cause disease, and (c) that most host cells have relatively limited need to proliferate at infected sites, since the exceptions often reveal evidence of the pathogen-host evolutionary arms race, as briefly addressed below. Indeed, many medically important infections involve exceptions to these generalizations where the host cannot take advantage of the stress vulnerability gap.

### 2.5 Pathogens have defenses against stress

There are four universal strategies for responding to stress:

1. Ignore the stress. Assume the stress is inconsequential or will soon pass.
2. Actively oppose or neutralize the stress.
3. Reduce metabolism / go dormant until the stress passes.
4. Leave to seek less stressful conditions elsewhere.
5. Immune-stressing, i.e., capitalizing on the stress vulnerability gap, is primarily aimed at pathogens using the first strategy—ignoring the stress and continuing with growth and synthesis as before. Immune-stressing is most effective against proliferating pathogens, which are the most dangerous.
6. The second strategic response is taken by many medically important pathogens, having evolved ways of actively opposing and neutralizing the stress. This stress-neutralization can involve utilizing different metabolic pathways or countering the potentially stressful host defenses such as resource restriction, ROS, acidity, or heat. Nevertheless, the pathogens’ necessity of actively opposing host-induced stress entails extra costs compared to not experiencing any stress at all.
7. The strategy of reducing metabolic activity is an effective defense against both resource-limiting stress and damaging/noxious stress. Indeed, dormant pathogens are particularly difficult to eliminate, with bacterial spores and latent viruses being extreme examples.
8. The fourth response to stress is to seek less stressful conditions. This involves avoiding being localized or actively fleeing locally stressful conditions in search of better conditions for proliferation. The host defense strategy of immune-stressing recognizes that both the pathogens and the localized stressors will spread out unless contained. Containment involves not just keeping the pathogens from accessing new resources, but also confining the pathogens along with the stressful conditions. Containment of the stressful conditions also reduces self-harm to nearby tissues.

An additional pathogen defense against host-induced stress is to have a population with individuals predisposed toward each of the four responses to stress. Not only does this bet-hedging occur between individuals, but also within each individual there is likely some degree of bet-hedging in the predispositions among the four basic responses to stress, reflected in the relative degree of gene transcription relevant to each response. This array of strategies for responding to stress complicates pathogen control.

Pathogens may evolve to minimize the stress vulnerability gap by seeking to infect delicate or critical host organs too costly for the host to substantially self-stress (e.g., brain, heart, eyes, bone marrow). Pathogen evolution to infect non-localizable tissues such as blood would also minimize the stress vulnerability gap. An example of a host counter offense would be to try to localize the blood together with immune defenses, as occurs in the spleen. In turn, a pathogen counter defense to this would be to minimize passage of infected RBCs in the spleen, as by adhering to capillaries in the delicate and vital brain, a strategy used by *Plasmodium falciparum* in humans and *Babesia bovis* in cattle (30).

### 2.6 It is not always feasible or even possible to kill every pathogen

As noted, it is relatively easy to kill localized, vulnerable proliferating pathogens that ignore the host-induced stress. Thus, immune-stressing can promptly help reduce the immediate threat. However, resistant pathogens that take the strategies of dormancy and/or actively opposing the stress may lead to chronic infection. This detente with non-proliferating pathogens entails the long-term costs of vigilance, including chronic inflammation, as well as the risk of recrudescence.

Because of the harm of inflammation (self-stressing), it should be expected that immune responses would be finely tuned to recognize when to shift from inflammation to the low-stress conditions conducive to repair that involve host cell proliferation and matrix synthesis. The value of this fine-tuning is reflected in the immunology literature, where nearly as much attention is given to anti-inflammatory immune suppression to prevent self-harm as is given to pro-inflammatory pathogen control. Indeed, much of the current focus of immunometabolism centers on limiting immune responses via metabolic regulation of effector function.

## 3 Beyond nutritional immunity

Nutritional immunity is the well-accepted host defense that restricts access of pathogens to critical metal ions, primarily iron, zinc, and manganese, presumably by compartmentalization of the pathogens and/or the nutrients (21, 31, 32). This restriction occurs most intensely at the phagolysosome, but it even occurs systemically as part of the acute-phase response. Some researchers have extended this concept of nutritional stressing to include restriction of certain amino acids, notably arginine (33, 34), tryptophan (3, 35, 36), and of deoxynucleotide triphosphates for making DNA, as well as glucose for energy (36). The host can also direct toxic concentrations of the same metal ions against pathogens (21, 36, 37), perhaps by suddenly dumping toxic amounts on especially susceptible nutrient-deprived pathogens. At present, nutritional immunity seems to be a side branch of immunometabolism that is outside of the paradigms built around metabolic regulation of immune cell function.

**Figure 1:**
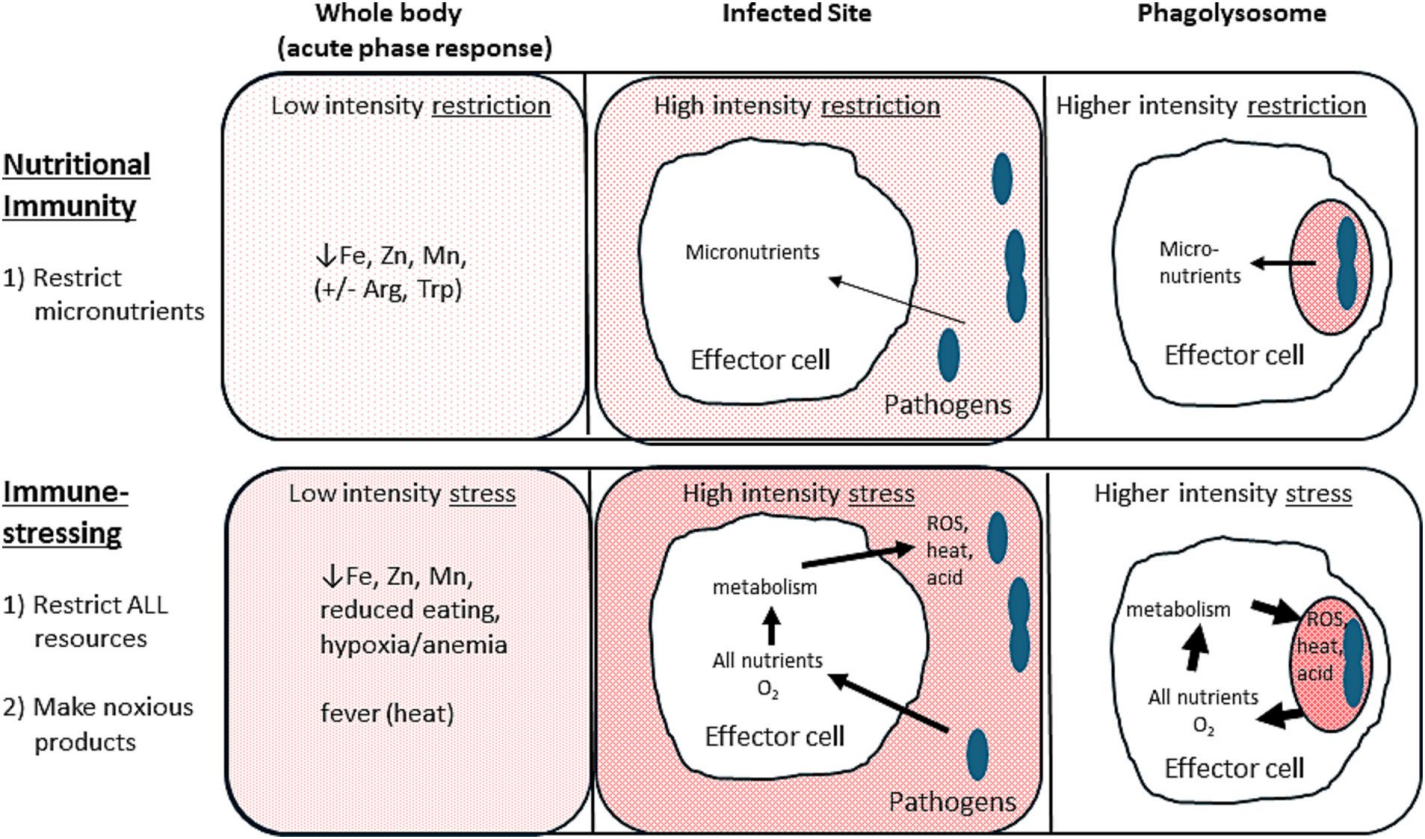
Nutritional immunity versus immune-stressing. Nutritional immunity (top portions) restricts micronutrients from pathogens. Immune-stressing (bottom portions) restricts all resources, including oxygen, and converts them to noxious metabolic products to further stress the pathogens. Arrows indicate the host-induced flow of resources and/or metabolic products to impair the pathogens. Arrow width and background shading intensity denote the relative degree of stress applied to the pathogens. Note that the more localized the pathogens (e.g., in the phagolysosomes) the more intense the stress that the host can safely apply.

Immune-stressing is the extension of nutritional immunity taken to its logical conclusion (Figure 1). All resources needed by pathogens, especially glucose and oxygen, should be restricted. Additionally, where feasible, those resources should be converted to noxious or damaging stressors. For glucose the main noxious stressor is lactic acid (38–41), while the noxious “waste” products of oxygen are ROS and heat. Once one recognizes the utility of stress preferentially applied to the pathogens, many of the conundrums in immunometabolism vanish. Therein, immune-stressing (encompassing and extending nutritional immunity) becomes the main trunk of immunometabolism, and metabolism’s key role in effector cell-pathogen interactions can be seen as controlling pathogens rather than controlling the immune response.

## 4 Caveats on activation phases

Immune-stressing as a strategy depends on effector cells’ own stress-vulnerable activities of proliferation and post-proliferative synthesis being completed *prior to* confronting the proliferating pathogens. Only simple priming (e.g., loading the weapon, rather than making the weapon) should be needed (42). Notable is that phagocyte NADPH oxidase has pre-synthesized components for generating ROS via the oxidative/respiratory burst that are kept separate until needed (43).

However, in much of the immunometabolism literature the distinction between activation for proliferation versus activation for effector function has not been made, such as with the assertions of high nutritional needs of effector cells for proliferation, synthesis, *and effector function*. This overlooks the basic vulnerability of proliferation. Another reason for the failure to make the distinction between effector cells’ proliferation (including post-proliferative synthesis) versus effector function is that both phases use enhanced or relatively increased aerobic glycolysis over OXPHOS and do indeed take up large amounts of resources. However, immune-stressing does not address the Warburg effect, the preferential use of aerobic glycolysis by rapidly proliferating cancer and effector cells, since the potential functions of aerobic glycolysis during proliferation (44–48) versus aerobic glycolysis while attacking pathogens are completely different. Immune-stressing applies to and emphasizes non-proliferating effector cells taking in large amounts of resources to keep them from the pathogens and converting resources to noxious waste, as occurs with glycolysis. The use of “Warburg” or “Warburg-like” for the glycolysis of effector cells confronting pathogens only adds to the confusion, as does the unqualified use of “effector cell activation.”

## 5 Specific resources and metabolic products

Immune-stressing potentially applies to all resources needed by pathogens as well as to all non-specific stressors generated by effector cells. These resources are virtually identical for host cells and pathogens, and currently a major theme in immunometabolism is that pathogens harm effector cells by competing for the same resources. Resources can be materials for cellular components (micronutrients, such as metal ions and vitamins, and macronutrients), or resources can be fuels for energy, such as macronutrients and oxygen. Table 2 lists potential resources and related noxious products and how they may play a role in immune-stressing. In this section special attention is given to glucose, glutamine, and oxygen as resources, and to lactic acid, ROS, heat, and acetate as stressful metabolic products.

**Table 2:** Resources as cellular components and fuels and their potentially noxious products.

Resources: cellular components	Potentially noxious products
Metal ions (e.g., Fe, Zn, Mn)	High concentrations can be toxic
Other minerals^1^	?
Vitamins^1^	?
Carbohydrates (esp. glucose)	Acids (lactic^2^, likely acetic^2^)
Amino acids	? (Ammonia?)
Glutamine	Ammonia?
Arginine	Nitric oxide
Tryptophan	Picolinic acid
Lipids	Antimicrobial lipids^2^? Acetic acid^2^?
Nucleic acids	?

Resources: fuels/energy	Potentially noxious products
Oxygen	ROS, heat
Carbohydrates (esp. glucose)	Acids (lactic^2^, likely acetic^2^)
Amino acids^2^	? (Ammonia?)
Glutamine	Ammonia?
Arginine	Nitric oxide
Tryptophan	Picolinic acid
Lipids^2^	Antimicrobial lipids^2^? Acetic acid^2^?

1 Not known to be specifically withheld from pathogens.

2 Restricted use as fuel without oxygen.

See text for discussion and references.

### 5.1 Glucose, glutamine and lactic acid

Glucose is the primary nutrient source for energy, and it has a central role in biosynthesis. Glutamine can also be an important energy source and plays a key role in nitrogen metabolism. The high rates of glycolysis and glutaminolysis by effector cells activated to attack pathogens present a conundrum, as stated by Curi, et al. (15):

*“The lymphocytes under study are not rapidly dividing, but possess the potential for cell division, the macrophages are terminally differentiated, and the neutrophils have a lifespan of approximately 10 h. Hence any hypothesis must explain high rates of fuel utilization in cells with widely different characteristics.”*

The explanation for the nutritional inefficiency that was developed by these authors was termed “branched-point sensitivity”—having large amounts of glucose and glutamine and their intermediates priming the numerous metabolic pathways to be ready for a sudden (i.e., unforeseen) metabolic response (49). More recent explanations for the high uptake of nutrients upon activation *to confront pathogens* are simply that the high costs are evidence that confronting pathogens must be metabolically costly. Glucose use via glycolysis, despite its inefficiency and wastefulness, is often considered preferential to OXPHOS because glycolysis produces ATP much faster than does OXPHOS (3, 45). These explanations presuppose that the metabolic needs of effector cells carrying out their sole function of controlling pathogens are unexpected and sudden. It has been noted that ATP production via glycolysis requires less cellular machinery than reliance on OXPHOS and hence is actually efficient if there is an abundance of glucose (45)—definitely not the case at infected sites. Others have proposed that glycolysis spares oxygen for use in generating antimicrobial ROS in the oxidative burst (50), a point to be addressed subsequently.

In sharp contrast, immune-stressing notes that effector cells use aerobic glycolysis because: (1) it is actively wasteful (~18x more wasteful than OXPHOS) to deprive proliferating pathogens of glucose, and (2) the lactate/lactic acid generated is a noxious waste product causing damaging stress (38–41), particularly to the more vulnerable pathogens. Note that the substantial energy value of lactate/lactic acid is not lost to the host since distant well-oxygenated tissues can use it for fuel. Speed of ATP generation is not important since the effector cells are not suddenly surprised by having to attack pathogens—that *is* their function. If energy were so critical, their OXPHOS pathways would presumably also have been well developed. Instead, evidence suggests that confronting pathogens is much less energetically costly than is typically assumed. It has been calculated that remarkably little energy is needed for cell motility (51), as would be needed for migrating toward pathogens. Furthermore, studies evaluating neutrophil energetics failed to find substantial increases in glucose uptake or lactate production immediately before or during phagocytosis, with researchers concluding that “*it seems safe to conclude that the rate of formation of ATP from carbohydrates is not increased during phagocytosis* (13).”

While the large amounts of glucose taken in by effector cells confronting pathogens are converted to and released as noxious lactate/lactic acid, the large amounts of glutamine taken up apparently in excess of their needs are converted to glutamate, aspartate, alanine, and lactate (15, 49). Besides “branched-point sensitivity” (52), other explanations for high glutamine uptake are based on the importance of maintaining the Krebs cycle for biosynthesis (though irrelevant at the infected site) and immune signaling (53, 54) and for generating noxious nitric oxide via arginine synthesis (3). In contrast, immune-stressing proposes that high glutamine uptake by effector cells actively depletes a major nutrient for pathogens, and also that the metabolic products should be put to good use. Conversion of glutamine (having two nitrogen atoms) to other amino acids yields ammonia. If used effectively for host-defense, speculatively the alkaline ammonia could protect effector cells from their own acids, and/or it could be very locally directed against the pathogens which are likely vulnerably primed to defend against acidosis.

### 5.2 Oxygen

With immunometabolism’s emphasis on high glucose usage via glycolysis by effector cells at infected sites, oxygen takes on a relatively minor role. Of course, oxygen is well recognized to be needed in the oxidative (or respiratory) burst by neutrophils and M1 macrophages to generate ROS to help kill pathogens. The oxidative burst serves not only to generate toxic ROS as a pathogen-damaging stressor, but it also depletes oxygen, thus serving as a resource-restricting stressor. Oxygen depletion at infected sites means that pathogens’ energy needs are mostly limited to glycolysis and fermentation, thus impairing their use of lactate, acetate (55), lipids, and amino acids for energy. An experimental finding strongly supporting immune-stressing as a strategy is that neutrophils (having few mitochondria) actively deplete oxygen from the tissues as they migrate toward the infected site (56). This, plus the finding that this oxygen is converted to noxious ROS, fits exactly with the predictions of immune-stressing—resource depletion coupled with noxious waste generation.

### 5.3 Heat

Immune-stressing proposes that resources to be kept from pathogens, such as oxygen and oxidizable fuels, should be converted to noxious or disruptive stressors, such as heat. Heat is *the* ignored metabolic product in immunometabolism, although the heat of fever is important in immunology. It noteworthy that fever is a *systemic* host-induced defense involving the slight core temperature increase of 1-4°C (38–41°C from 37°C in humans) (57). However, lethal temperatures for mammalian cells, and presumably most pathogens that infect them, are around 45°C given enough time, with even a single degree or two above this greatly increasing the kill rate (58). Of course, a key point of this conceptual analysis is that the synthesis involved in proliferation is particularly susceptible to damaging stressors such as heat. As suggested in the section on pathogen defenses against immune-stressing, dormant pathogens and/or those opposing heat stress via the heat shock response should be much more heat-tolerant.

Fever, like all systemic stressors of the acute phase response, must necessarily be only mildly stressful compared to the more intense stress that can be safely applied locally at the infected site, and especially ultra-locally within the phagolysosome (Figure 1) (2). Strangely, the benefits and sources of intense localized heat have only rarely been considered (2, 29). Since substantial heat is generated by the oxidative burst via phagocyte NADPH oxidase (22, 23), it has been argued that the systemic heat of fever functions to raise the ambient temperature to aid this intensely localized heat produced at the surface of pathogens (29). While these temperatures have never been measured, substantial highly localized heat is also generated in OXPHOS, with mitochondrial temperatures of 50°C detected using heat-sensitive dyes (59). Recent work has confirmed that mitochondrial temperatures are adapted to be as much as 15°C higher than core body temperatures (60), meaning that much higher temperatures than previously recognized can be locally generated by oxidation. Interestingly, mitochondria have been shown to be recruited to macrophage phagosomes to help kill bacteria by augmenting ROS generation (61), though the associated heat was not mentioned (or considered?).

Heat as a damaging/noxious stressor can be synergistic with other damaging stressors such as acidity and ROS (62) as well as with iron restriction (63). The standard view of the oxidative burst is that the entire antimicrobial effect comes from ROS, ignoring not only oxygen depletion but also the extremely localized heat generation. The oxidative burst also releases arachidonic acid, which causes lipid peroxidative damage to bacteria (64), whose effects are likely enhanced by synergy with this heat.

### 5.4 Acetate/acetic acid

It was recently found that infections and inflammatory conditions can increase acetate concentrations 5x above normal (1 mM) in the blood (65) and 100x above normal at inflamed foci (55). The rising local acetate levels were found to help control infections early on by stimulating effector lymphocyte function, while later the high acetate concentrations became immunosuppressive. The authors described specific mechanisms by which acetate functions as a context-sensitive metabolite to control infection and also to limit inflammatory damage (55). The source of the acetate was not determined.

In immune-stressing this important finding of acetate abundance at inflamed sites is readily interpreted as a noxious metabolic product, comparable to and complementary with lactic acid. Such high local acetate/acetic acid concentrations (100 mM) can be inhibitory or lethal to bacteria (66, 67). Growth inhibitory activity of acetate against *Pseudomonas* was found to be synergistic with low pH, with substantial inhibition at 20 mM at pH 6 (68). Acetate at 12.5 mM for 24 h killed half of the neoplastic thymocytes tested, while lacking apparent cytotoxicity in normal thymocytes (69) (which presumably were not actively proliferating). Like lactate/lactic acid, acetate’s toxicity is due to its high local concentration; and its use for energy at infected sites is likely limited by local hypoxia, while still being readily usable as fuel by distant host cells. The 5x increase in blood acetate levels is in line with other systemic stresses of the acute phase response—they are necessarily much milder than the local stresses, but they enhance the gradient of the stress in addition to making conditions somewhat less favorable for the proliferation of pathogens which have escaped the intense local stresses (2). The enhanced effector cell functionality of slightly increased acetate concentrations (55) parallels that of other stressful metabolic conditions such as slight hypoxia, acidity, and even heat, where it is reasonable that effector cells evolved to function best in their expected working conditions, the somewhat stressful conditions that they themselves have created (29). It will be interesting to determine the metabolic sources of the high acetate concentrations at inflamed sites and the cells responsible (presumably the effector cells themselves).

## 6 Glycogen storage

### 6.1 A test of paradigms

Given the importance of glucose at infected sites, it is not surprising that effector cells store glucose as glycogen and bring it with them as they attack the pathogens (70–72). However, the standard paradigm of immunometabolism differs from immune-stressing in a testable prediction. The standard paradigm notes that glucose is so important for effector cells’ function that they should store as much glucose (as glycogen) as practical and carry it with them to the infected site (73). Although not stated, the implication is that effector cells should fill up with glycogen while still in the blood where glucose is plentiful, rather than relying on taking up diminishing amounts of glucose upon approaching the infected site.

Immune-stressing, as a strategy, emphasizes that glucose is especially important for the proliferating pathogens, but only somewhat important to the non-proliferating effector immune cells’ metabolic needs. Therefore, the effector cells should deplete as much glucose from the tissues as practical and store it away as glycogen. This extra glucose brought into the infected site, which tends to be used last (13), can also be converted later to additional lactic acid as noxious waste to further stress the pathogens. Importantly, the effector cells should *not* fill up with stored glucose (glycogen) while in the blood. Rather, they should vigorously take up glucose as they migrate to the infected site to create a zone of locally increasing stress to harm the more vulnerable pathogens. This means that while late arriving effector cells may not become replete with glycogen, they will still be helping deplete the glucose that the pathogens especially need.

### 6.2 Experimental results

It has been shown that effector cells, most notably neutrophils, take up glucose for storage as glycogen predominantly *after* they leave the blood and are migrating to the infected site (70–72). This finding is counter to that implied by the standard paradigm, but it is in line with immune-stressing which proposes that glycogen storage in route to the infected site allows for even greater stress (less glucose and more lactic acid) to be directed against the pathogens.

## 7 Metabolism and immunosuppression

Effector cells lose functionality when exposed to especially stressful conditions, primarily low glucose (74), but also other nutrient deficiencies and extreme hypoxia (56, 75) and acidity (76). In these very stressful conditions, immunosuppression with reliance on OXPHOS takes over and leads to tissue repair. Bacterial fermentation products such as short chain fatty acids, as would develop in stressful oxygen-depleted sites, can also be immunosuppressive (77, 78).

Resource depletion and waste buildup at infected sites are typically viewed in immunometabolism (and immunology in general) as the expected byproduct of the struggle for resources between immune cells and pathogens. In this view it is the vulnerability of effector cells to increasing stress that largely allows metabolism to regulate or control effector cell responses. In contrast, immune-stressing considers this stressful environment not as a simple byproduct of the metabolism of conflicting pathogens and host cells, but as an active host defense to preferentially harm the more vulnerable proliferating pathogens. The increasing stresses at infected sites are seen as cues to induce the switch from damaging pro-inflammatory conditions to anti-inflammatory and tissue repair conditions, either because the pathogens have been controlled or because they must be tolerated as a chronic infection.

A major concern in immunometabolism is that the microenvironment around many cancers is immunosuppressive due to the tumor cells outcompeting effector cells for resources and accumulation of noxious wastes. Not only are effector cells functionally impaired, but the surrounding immune cells are immunosuppressive and may actively promote tumor growth (9, 73, 74, 79–81). The inability of effector cells to control these tumors has been considered an immune dysregulation, and much of the emphasis in immunometabolism is directed at correcting this metabolic dysfunction (79, 82).

An alternative interpretation presented here is that a large enough assemblage of tumor cells, having already evaded immune control, can create stressful enough local conditions that effector cells misinterpret the situation as needing more immune suppression. A similar condition likely occurs with several infectious diseases such as cryptococcosis and lepromatous leprosy (83, 84), where early immune evasion or underlying immunosuppression allows accumulation of a large pathogen mass (the organisms themselves or infected macrophages), which then can cause metabolically stressful conditions that enhance the immunosuppression. Rather than being seen as immune dysregulation or dysfunction, this metabolic stress that enhances prior immune evasion can be seen as a pathogen subversion of the typically effective strategy of immune-stressing.

## 8 Discussion

The concept of immune-stressing offers a simple, yet powerful, explanation for almost any puzzle involving the depletion of nutrients at infected sites, as well as for the metabolism of those nutrients into noxious products to be directed against the more vulnerable localized proliferating pathogens. Not only does immune-stressing apply to the central conundrums of effector cells’ high glucose and glutamine uptake and inefficient glucose use via aerobic glycolysis with lactic acid production *while in conflict with pathogens*, but it emphasizes the value of oxygen depletion and the use of localized heat as host defenses. Immune-stressing thereby greatly expands the protective value of the oxidative burst beyond simply ROS production. Immune-stressing is compatible with the finding of neutrophil secretory granules having glutaminase (85) and arginase I, which likely act to deplete the phagolysosomes of these amino acids to stress the pathogens (33, 34). Arginine can also be depleted to produce toxic nitric oxide, and tryptophan is well known to be depleted by effector cells (3). Picolinic acid, a secondary metabolite of tryptophan, has antimicrobial activity (86). Lipid metabolism by effector cells at infected sites should also be reconsidered in light of immune-stressing. For example, there is high uptake of lipid by neutrophils moving from the blood toward infected sites (70), and there is prominent synthesis of fatty acids from citrate stored in LPS-stimulated macrophages and other effector cells (3). Immune-stressing would interpret these findings as attempts to remove nutrients from the pathogens, and where feasible to convert them to toxic byproducts such as antimicrobial lipids (87, 88) or acetate, whose metabolic source at inflamed sites is yet undetermined (55).

While the potential scope of immunometabolism is vast, covering all interactions of nutrition, metabolism, and immunology from the systemic to the subcellular levels, unfortunately immunometabolism as a distinct term became applied much more narrowly to the metabolism of immune cells. Surprisingly, the immune system’s use of metabolism for control of pathogens is seldom mentioned (2, 36). Since the basic function of the immune system is to control pathogens, it is proposed that effector cells evolved to account for not only their own metabolic needs, but especially the metabolic needs of the even more vulnerable proliferating pathogens. For most non-storage cells it would seem obvious that high nutrient uptake reflects high metabolic needs and therefore these needs must be nutritionally costly. However, this is not true for effector cells actively confronting pathogens because of the genetic conflict with the pathogens. In immune-stressing the high nutrient uptake is to deprive the pathogens and to subject them to the noxious “waste” products. Understanding the acute phase response and its myriad systemic metabolic changes during infections (e.g., metal ion restriction, heat/fever, anorexia) requires recognition of the pathogens’ vulnerability to these metabolic changes (2). Likewise at the cellular level, the pathogens’ metabolic needs must be considered to understand the metabolism of the local immune response. It is argued that the current narrow field of immunometabolism has been led astray by emphasizing control of immune responses rather than the basic function of the immune system in controlling pathogens. By ignoring the vulnerability of pathogen proliferation to stress, the use of glycolysis and the high uptake of glucose and glutamine by effector cells has been misinterpreted. In contrast, the immune-stressing concept emphasizes that proliferating pathogens that are localized are especially vulnerable to even completely non-specific stress. Recognition of this auxiliary function of effector cells to apply stress to pathogens has profound implications, many of which suggest new paradigms for interpreting metabolic findings relating to effector immune function. It is proposed that immunometabolism should be the study of how the immune system uses nutrition and metabolism to help control pathogens and to help assess metabolic cues for guiding the appropriateness of the response.

This conceptual analysis emphasizing how immune cells use metabolism to control pathogens leads to numerous research questions, some of which would never have been considered worthwhile or even recognized for addressing. For instance, what other resources (particularly micro- and macronutrients beyond those addressed earlier) are also actively restricted by the infected host? More interestingly, how can each of the restricted resources be converted to damaging/noxious stressors to harm the more vulnerable pathogens? Specifically, is the ammonia derived from glutaminolysis put to good use by effector immune cells to capitalize on the stress vulnerability gap, and if so, how? Which cells are responsible for the noxious concentrations of acetate/acetic acid at infected sites; which nutrients are metabolized to the acetate/acetic acid; and are the relative concentrations of lactate/lactic acid and acetate/acetic acid at infected sites adjusted by the effector cells to maximize the stress vulnerability gap? Are the lipids and lipid precursors which are actively taken up by effector cells that approach infected sites metabolized to create noxious waste (e.g., acetic acid, antimicrobial lipids)? What degree of hypoxia is needed at infected sites to effectively bar pathogens from oxidizing nutrients for energy (e.g., lactate, acetate, lipids, and amino acids)? A glaring need is to determine the actual temperatures to which pathogens are exposed, particularly the surface of phagocytized pathogens exposed to the oxidative burst. Exactly how hot is it directly at the site of ROS generation, recognizing that the surface of a furnace is markedly hotter than the house it is heating? And what are the actual nutrient needs and energy costs of effector immune cells as they confront pathogens, recognizing that their resource uptake at infected sites is not a reliable indicator of their own metabolic needs?

Exploration of the vulnerabilities of pathogens to localization and proliferation, contrasting with their well-recognized advantages of rapid evolution, challenges or creates numerous basic paradigms of immunometabolism and even immunology. New paradigms lead to new insights and generate questions and findings never even imagined. So it is with immune-stressing.
